# Synthesis and Electron-Transporting Properties of N-Type Polymers with Cardanol-Based Side Chains

**DOI:** 10.3390/mi15121475

**Published:** 2024-12-05

**Authors:** Jin Cheng, Hongyu Fu, Yifan Qiao, Xuming Xue, Pirada Sudprasert, Kenji Ogino

**Affiliations:** 1 Department of Chemical Engineering and Pharmaceutical Engineering, Changzhou Vocational Institute of Engineering, Changzhou 213164, China; 2 Graduate School of Bio-Applications and Systems Engineering, Tokyo University of Agriculture and Technology, Tokyo 184-8588, Japan; 3 Faculty of Science and Technology, Rajamangala University of Technology Tawan-OK, Chonburi 20110, Thailand

**Keywords:** cardanol-based, n-type polymer, electron-transporting properties

## Abstract

We synthesized n-type polymers poly{[N,N′-bis(2-octyldodecyl)-naphthalene-1,4,5,8-bis(dicarboximide)-2,6-diyl]-alt-5,5′-(2,2′-bithiophene)} [P(NDI2OD-T2)] and poly{[N,N′-bis(3-(4-cardanol)propyl)-naphthalene-1,4,5,8-tetracarboxylic diimide]-alt-[5,5′-bis(2-thienyl)-2,2′-bithiophene]} [P(NDICL-T2)] with cardanol-based side chains via Stille coupling to enhance electron mobility. Replacing the 2-octyldodecyl side chain with cardanol in P(NDICL-T2) improved electron mobility due to increased chain flexibility and ordered packing. Lower glass transition temperature (*t*_g_), red-shifted UV-vis absorption, results from crystalline structure analysis, indicating tighter lamellar spacing and enhanced molecular ordering, and smoother surface morphology confirmed the enhanced intermolecular interactions and uniform film formation.

## 1. Introduction

The development of high-performance n-type polymers for organic electronic devices has garnered significant attention due to their potential applications in organic field-effect transistors (OFETs), organic photovoltaics (OPVs), and other electronic devices [1,2]. Among the various n-type polymers, poly{naphthalene-1,4,5,8-bis(dicarboximide)} (pNDI) derivatives have emerged as highly promising candidates [3,4,5,6,7,8]. A notable example is poly{[N,N′-bis(2-octyldodecyl)-naphthalene-1,4,5,8-bis(dicarboximide)-2,6-diyl]-alt-5,5′-(2,2′-bithiophene)} [P(NDI2OD-T2)], which has garnered significant attention for its excellent solubility and processability in solution. These properties are largely attributed to the introduction of solubilizing alkyl chains into the nitrogen atoms of the polymer backbone [9]. Additionally, P(NDI2OD-T2) demonstrates high electron affinity and favorable energy levels, derived from the naphthalene diimide (NDI) moieties [10]. As a result, considerable efforts have been made to further enhance the electron-transporting performance of polymers based on P(NDI2OD-T2).

Nevertheless, the relatively large dihedral angle between the naphthalene-1,4,5,8-bis(dicarboximide) (NDI) unit and adjacent thiophene units poses a challenge to achieving high coplanarity in the polymer backbone, limiting device performance enhancement [11]. While the NDI unit itself is planar, one promising approach to address these challenges is the engineering of side chains, which can profoundly influence the polymer’s solubility, crystallinity, and overall packing behavior [12,13,14,15,16]. The selection of an appropriate side chain is critical, as it can enhance the polymer’s morphology, improving its electronic properties. In this context, cardanol, a phenolic lipid derived from cashew nut shell liquid (CNSL), has emerged as an intriguing candidate for side-chain engineering. The motivation for using cardanol lies in its unique structure: the long, flexible alkyl chain with an aromatic ring offers the potential for improving molecular packing and reducing steric hindrance, which are key factors in enhancing electron mobility [17,18,19].

This work synthesized P(NDI) derivatives with cardanol-based side chains by introducing cardanol groups into the NDI side chains. The polymerization process yielded a polymer with substitution of the original 2-octyldodecyl (2OD) side chains, resulting in poly{[N,N′-bis(3-(4-cardanol)propyl)-naphthalene-1,4,5,8-tetracarboxylic diimide]-alt-[5,5′-bis(2-thienyl)-2,2′-bithiophene]} [P(NDICL-T2)]. The structure of this polymer was confirmed using proton nuclear magnetic resonance (^1^H-NMR) spectroscopy and gel permeation chromatography (GPC). The thermal, optical, morphological, and crystalline properties of the modified polymer were then compared with those of the unmodified P(NDI2OD-T2) to evaluate the impact of cardanol group incorporation. Additionally, the electron mobility of devices based on this polymer was measured to reveal the effects of cardanol introduction on device performance.

## 2. Materials and Methods

### 2.1. Materials

P(NDI2OD-T2) was synthesized according to the established procedure [20]. Cardanol (CL-1), 1,3-dibromopropane, dimethylformamide (DMF), potassium phthalimide, hydrazine hydrate, 2,6-dibromo-1,4,5,8-naphthalenetetracarboxylic dianhydrid (NDA-Br), o-xylene, propionic acid, 5,5-bis(trimethylstannyl)-2,2′-bithiophene (T2), PdCl_2_(PPh_3_)_2_, 2,6-dibromo-1,4,5,8-naphthalenetetracarboxylic di(2-octyldodecyl)imide (NDI2OD-Br), 2-bromothiophene, and other reagents were commercial products used without further purification.

### 2.2. Synthesis of 3-Bromopropyl Substituted Cardanol (CL-2)

A 150 mL round-bottom flask was charged with CL-1 (10.08 g, 34 mmol), 1,3-dibromopropane (10.10 g, 50 mmol), K_2_CO_3_ (6.90 g, 50 mmol), and DMF (75 mL). The mixture was stirred at room temperature for 48 h. Upon completion of the reaction, the mixture was subjected to ultrasonic agitation at 50–60 °C for 12 h. The DMF was removed under reduced pressure using a rotary evaporator. The resulting residue was diluted with ethyl acetate and washed successively with water, 5 M NaOH solution, and saturated brine. The organic layer was dried over anhydrous MgSO_4_, followed by filtration and concentration under reduced pressure. The crude product was then purified by column chromatography using a mixture of ethyl acetate and n-hexane (1:10) as the eluent. A light-yellow viscous liquid, CL-2, was obtained with a yield of 10.20 g, corresponding to a 72% yield. The ^1^H-NMR spectrum was shown in Figure S1.

### 2.3. Synthesis of 3-Phthalimidopropyl Substituted Cardanol (CL-3)

In a 100 mL round-bottom flask, CL-2 (8.47 g, 20 mmol) was combined with potassium phthalimide (1.62 g, 21.9 mmol) and DMF (22 mL). The mixture was stirred at 90 °C for 6 h. After the reaction was complete, the mixture was allowed to cool to room temperature. To the reaction mixture, 180 mL of distilled water and 95 mL of dichloromethane were added, and the solution was extracted three times. The combined organic layers were then treated with 0.2 M KOH solution and washed with saturated NH_4_Cl solution. The organic phase was dried over anhydrous Na_2_SO_4_ and concentrated under reduced pressure. The crude product was purified via column chromatography using a dichloromethane/n-hexane mixture (1:4) as the eluent. The purified product, CL-3, also a light-yellow viscous liquid, was obtained in a yield of 8.02 g, representing an 82% yield. The ^1^H-NMR spectrum was shown in Figure S2.

### 2.4. Synthesis of 3-Aminopropyl Substituted Cardanol (CL-4)

A 50 mL round-bottom flask was charged with CL-3 (7.34 g, 15 mmol) and 34 mL of methanol. The mixture was stirred, and hydrazine hydrate (2.0 mL) was then added. The reaction setup was assembled and checked for airtightness, followed by heating under reflux for 8 h. After it was cooled to room temperature, the solvent was removed under reduced pressure. The residue was dissolved in 400 mL of dichloromethane, and the solution was washed twice with 2 M KOH solution. The organic layer was then washed twice with 200 mL of saturated brine solution. After it was dried over anhydrous Na_2_SO_4_, the solution was filtered and concentrated under reduced pressure, yielding 4.63 g of a light yellow viscous liquid product, CL-4, with an 86% yield. The ^1^H-NMR spectrum was shown in Figure S3.

### 2.5. Synthesis of 2,6-Dibromo-1,4,5,8-Naphthalenetetracarboxylic Di(3-(4-Cardanol)Propyl)Imide (NDICL-Br)

In a 50 mL round-bottom flask, NDA-Br (0.94 g, 2.2 mmol), CL-4 (3.60 g, 10 mmol), o-xylene (20 mL), and propionic acid (2.5 mL) were combined. The mixture was stirred at 140 °C for 4 h and then allowed to cool to room temperature. The solvent was removed under reduced pressure. The crude product was purified by column chromatography using a mixture of ethyl acetate and n-hexane (1:4) as the eluent, yielding a red solid product, NDICL-Br (1.74 g, 71% yield). The ^1^H-NMR spectrum was shown in Figure S4.

### 2.6. Synthesis of P(NDI2OD-T2)

A three-neck flask was equipped with a magnetic stir bar and setup under a nitrogen atmosphere. The flask was purged with nitrogen and dried with a heat gun for over 10 min to ensure it was completely dry. Under nitrogen protection, NDI2OD-Br (0.50 g, 0.50 mmol), T2 (0.27 g, 0.55 mmol), and PdCl_2_(PPh_3_)_2_ (6.6 mg, 0.016 mmol) were added to the flask, followed by the addition of anhydrous toluene (15 mL) after nitrogen replacement. The reaction mixture was subjected to three freeze–thaw cycles to degas the solution with liquid nitrogen. The reaction mixture was stirred at 90 °C for 96 h, after which 2-bromothiophene (0.25 mL, 4.1 mmol) was added. Stirring was continued at 90 °C for an additional 12 h, and after cooling to room temperature, KF (2.76 g, 79.2 mmol) was added. The mixture was stirred for 2 h before allowing the reaction to cool to room temperature. The reaction mixture was washed twice with 150 mL of chloroform, and the combined organic layers were concentrated and reprecipitated in methanol, leading to the formation of a dark green flocculent solid. The suspension was filtered, which was then purified by Soxhlet extraction with acetone, ethyl acetate, and hexane until the solution was colorless. The solid was then collected via Soxhlet extraction with CHCl_3_ and reprecipitated in methanol to yield the final polymer product. The product was obtained as a dark-blue powder, weighing 0.43 g, with a yield of 86%. The ^1^H-NMR spectrum and GPC plots were shown in Figure S5 and Figure S7  respectively.

### 2.7. Synthesis of P(NDICL-T2)

The procedure for synthesizing P(NDICL-T2) was similar to that for P(NDI2OD-T2), with adjustments to the monomer composition. In this synthesis, the original NDI2OD-Br (0.50 g, 0.50 mmol) was substituted with cardanol-based NDICL-Br (0.50 g, 0.50 mmol). The resulting product was obtained as a dark-green powder with a yield of 69% (0.38 g). The ^1^H-NMR spectrum and GPC plots were shown in Figure S6 and Figure S7  separately. The characteristics of P(NDI2OD-T2) and P(NDICL-T2) are summarized in Table 1.

### 2.8. Characterizations

^1^H-NMR spectra were recorded on a Bruker 600 MHz instrument at 25 °C, using deuterated chloroform as the solvent and tetramethylsilane as the internal standard.

The number-average molecular weight (*M*_n_) and polydispersity index (PDI) were determined by gel permeation chromatography (GPC) with an Agilent 1260 detector, using chloroform as the eluent at 0.5 mL/min.

Glass transition temperature (*t*_g_) was measured by differential scanning calorimetry (DSC) on a Rigaku DSC-8230 under nitrogen, with heating and cooling rates of 10 °C/min.

Ultraviolet-visible (UV-vis) absorption spectra were obtained using a JASCO V-570 spectrophotometer.

Film morphology was studied using atomic force microscopy (AFM) (SII Nanocute, Tokyo, Japan).

To investigate the crystal structures of films deposited on glass substrates, we utilized grazing incidence wide-angle X-ray diffraction (GIWAXD) with a RIGAKU SmartLab X-ray diffractometer. The instrument operated with Cu K_α_ radiation (λ = 1.5418 Å) at settings of 45 kV and 200 mA. Measurements were performed in the out-of-plane configuration, scanning from 2° to 35° with a step increment of 0.01° and a scanning speed of 1° per minute. The angle of incidence was maintained at 0.14°. The thicknesses of the films and metals were determined using a BRUKER Dektak XT-S stylus profilometer.

### 2.9. Electron-Only (EO) Devices Fabrication and Detection

Prior to the fabrication of EO devices, indium tin oxide (ITO) patterned glass substrates (10 Ω/square) underwent cleaning using an alkaline detergent with ultrasonic agitation, followed by thorough rinsing with deionized water. The substrates were then subjected to ultrasonic cleaning in 2-propanol, rinsed again with fresh 2-propanol, and dried under a stream of nitrogen gas. The EO devices were constructed with a layered structure of ITO/aluminum (Al) (50 nm)/active polymer layer: P(NDI2OD-T2) or P(NDICL-T2) (200 nm)/lithium fluoride (LiF) (0.5 nm)/Al (100 nm). A 50 nm-thick aluminum layer was deposited onto the ITO surface under vacuum conditions at 3.0 × 10^−4^ Pa. The active polymer layer was spin-coated onto the Al layer at 1000 rpm for 60 s from a chlorobenzene solution (40 mg/mL) passed through a 0.45 µm membrane filter. For the cathode, a 0.5 nm-thick LiF layer followed by a 100 nm-thick Al layer was deposited onto the polymer film under the same vacuum conditions, with deposition rates of 0.1 Å/s and 4.5 Å/s, respectively, using tantalum and tungsten boats. The photoactive area of each device was typically 6 mm^2^. Current-voltage measurements were carried out using a DC voltage and current source/monitor (Keithley 2400, Cleveland, OH, USA).

Electron mobility in the space-charge-limited current (SCLC) region was determined from current density-voltage and double logarithmic curves, with applying the following Equation (1) to account for series resistance (8 Ω) and assuming a near-zero built-in voltage [21].

(1) $$J=\frac{9}{8}{\varepsilon_{r}\varepsilon }_{0}\mu_{e}\frac{V^{2}}{L^{3}}$$

where *J* is the electron current density, mA/cm^2^; *μ*_e_ is the electron mobility, cm^2^/Vs; *ε*_r_ is the relative permittivity of the material (3.5), *ε*_0_ is the permittivity of vacuum (8.854 × 10^−14^ F/cm), *L* is the thickness of the polymer layer, cm; and *V* is the voltage drop across the device, V.

## 3. Results

### 3.1. Synthesis and Characterization

**Scheme 1:**
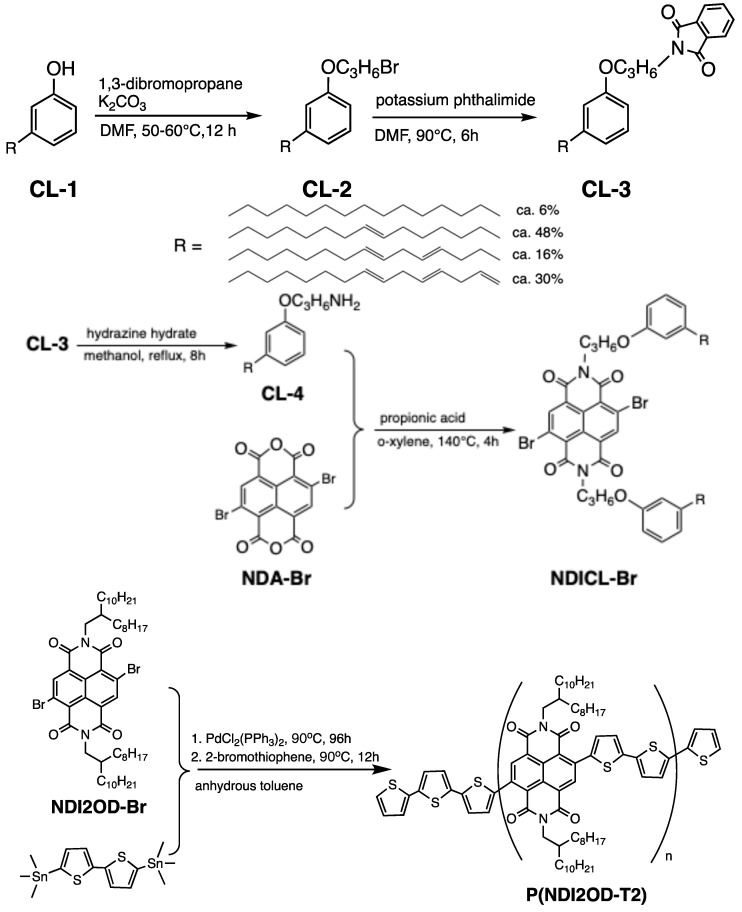

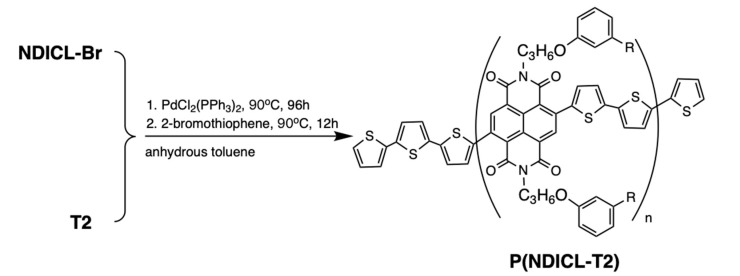
Synthesis routes.

In Scheme 1, we showed the synthesis routes of P(NDICL-T2), P(NDI2OD-T2), and P(NDICL-T2).

During synthesis, CL-1, a mixture with a stable and well-defined composition (as shown in Scheme 1 for the R group, and confirmed by ^1^H-NMR data), was utilized as a source for side-chain modification. Due to the consistent composition of CL-1, this mixture-based approach is expected to have minimal impact on chain packing and film morphology, thereby not affecting the reproducibility of electron-transporting properties.

The process began with the synthesis of cardanol derivatives (CL-2, CL-3, and CL-4), followed by the polymerization of NDI derivatives using Stille coupling reactions.

To modulate the properties of the resulting polymers, we synthesized two homopolymers, P(NDI2OD-T2) and P(NDICL-T2), by adjusting the side chains on the NDI core. Stille coupling, utilizing PdCl_2_(PPh_3_)_2_ as a catalyst, was employed to link NDI2OD-Br or NDICL-Br with T2, yielding polymers with high purity. The freeze–thaw cycles used during polymerization ensured thorough degassing of the reaction mixture, preventing side reactions, and promoting efficient polymer chain growth. The successful synthesis of P(NDI2OD-T2) and P(NDICL-T2) demonstrates the versatility of the polymerization process in creating polymers with distinct side-chain compositions.

The M_n_ and PDI of the synthesized polymers, P(NDI2OD-T2) and P(NDICL-T2), were characterized and are reported in Table 1. The M_n_ ranged from 27,553 g/mol for P(NDI2OD-T2) to 24,346 g/mol for P(NDICL-T2), with PDIs between 3.13 and 3.76. The differences in M_n_ and PDI between P(NDI2OD-T2) and P(NDICL-T2) reflect the influence of the side chains on polymerization control, likely due to variations in solubility and chain flexibility imparted by the 2OD and cardanol side chains.

### 3.2. Thermal Properties

**Figure 1:**
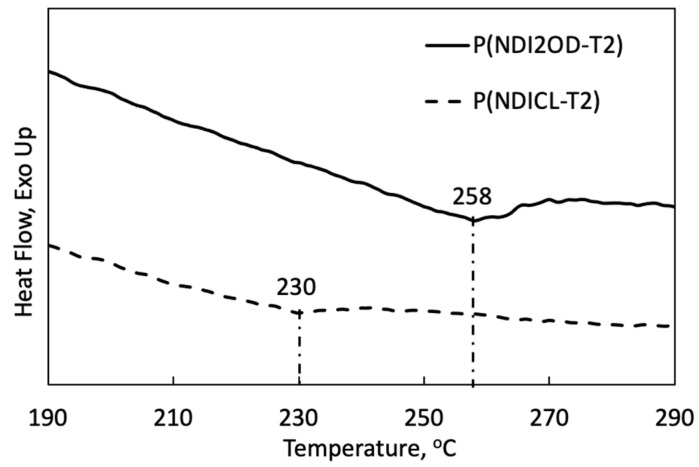
DSC thermograms for P(NDI2OD-T2) and P(NDICL-T2).

As depicted in Figure 1, the t_g_ of P(NDI2OD-T2) was observed at 258 °C, which decreased to 230 °C for P(NDICL-T2). This trend indicates that incorporating cardanol side chains increases the flexibility of the polymer chains, thereby lowering the glass transition temperature. Increased flexibility may improve the solubility and film-forming properties of conjugated polymers, potentially enhancing device performance [22]. On the other hand, this added flexibility can also affect n-π* stacking between polymer backbones, which may lead to variations in electron mobility [23].

### 3.3. Optical Properties

**Figure 2:**
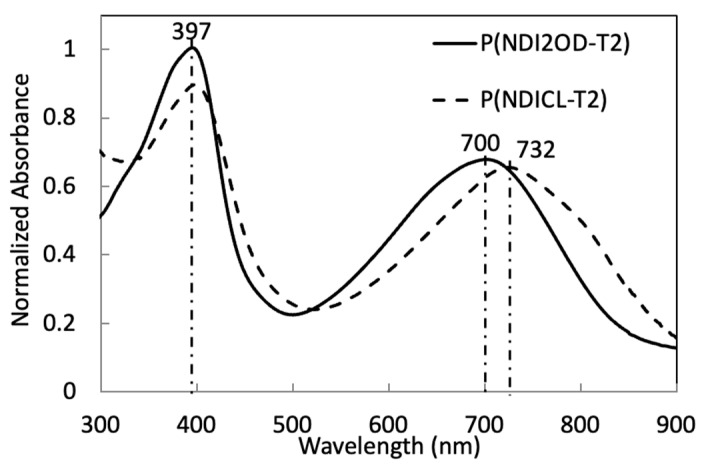
Film UV-vis spectra of P(NDI2OD-T2) and P(NDICL-T2). Films were spin-coated at 1000 rpm for 60 s from a 40 mg/mL chlorobenzene solution filtered through a 0.45 µm membrane filter, on 2.5 cm × 2.5 cm glass plates.

As shown in Figure 2 and Table 2, the polymer P(NDI2OD-T2) exhibits major absorption peaks at approximately 397 nm and 700 nm, corresponding to localized π→π* electronic transitions within the polymer units and n→π* transitions indicating charge transfer states, respectively [24]. P(NDICL-T2) also shows an absorption peak around 397 nm, suggesting a similar localized π→π* transition. However, in the longer-wavelength region, P(NDICL-T2) demonstrates a significant red shift to 732 nm.

This red shift, observed in the thin-film state, was likely due to more ordered π-π polymer packing, enabled by the cardanol substitution, which enhances intermolecular interactions and promotes charge transfer through n→π* transitions.

### 3.4. Morphological Properties

**Figure 3:**
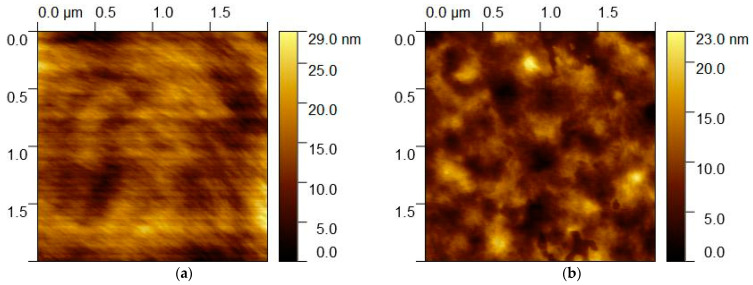
AFM height images (2 μm × 2 μm): (**a**) P(NDI2OD-T2) (RMS = 3.929 nm); (**b**) P(NDICL-T2) (RMS = 3.127 nm). Films were formed via the same procedure as UV-vis on 2.0 cm × 2.0 cm silicon probes (resonant frequency ≈ 300 kHz, force constant ≈ 26 N/m, Olympus OMCL-AC160TS).

The AFM topography images are depicted in Figure 3. Films of both polymers exhibited no obvious phase separation. The AFM measurements of P(NDI2OD-T2) and P(NDICL-T2) revealed root-mean-square roughnesses (RMS) of 3.929 nm and 3.127 nm, respectively. The decrease in RMS indicates that substituting 2OD with cardanol as a side chain results in a smoother film surface. This reduction in surface roughness suggests that cardanol improves the uniformity of the film, enhancing surface properties.

### 3.5. Crystalline Structure Analysis

**Figure 4:**
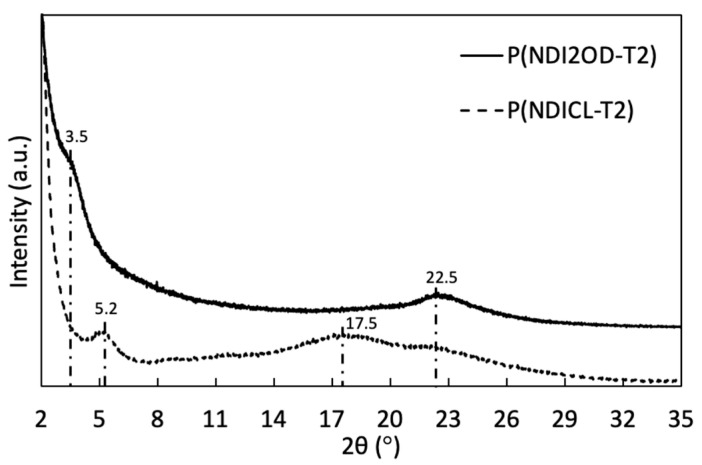
GIWAXD profiles were obtained with out-of-plane geometry for thin films of P(NDI2OD-T2) and P(NDICL-T2) fabricated with chlorobenzene.

Crystalline structure analysis using GIWAXD revealed distinct differences between P(NDI2OD-T2) and P(NDICL-T2), as shown in Figure 4. For P(NDI2OD-T2), diffraction peaks at 2θ ≈ 3.5° and 22.5° correspond to alkyl stacking in the edge-on orientation and π–π stacking distances in the face-on orientation, respectively [25]. In contrast, P(NDICL-T2) exhibited peaks at 2θ ≈ 5.2°, 17.5°, and 22.5°.

As shown in Table 3, the lattice spacing was calculated using Bragg’s law Equation (2),

(2) $$d=\frac{\lambda }{2sin\theta }$$

where *d* is the lattice spacing, *λ* the CuK_α_ wavelength, and *θ* the diffraction angle.

The shift in the lamellar peak from 3.5° to 5.2° suggests a reduced lamellar spacing due to substituting cardanol-based groups, allowing for tighter chain packing. The appearance of a new diffraction feature at 2θ ≈ 17.5°, corresponding to a d-spacing of approximately 5.07 Å, suggests enhanced lateral ordering in the polymer chains. While this feature is weak and broad, its position aligns with possible lateral chain packing or (020) reflections, likely influenced by the unique packing behavior of the cardanol side chains [26]. Although the broadness of the peak could indicate a partial disorder or strain within the lattice, it also highlights structural modifications introduced by the side chains. While the consistent π–π stacking peak at 22.5° in both polymers indicates that the interchain stacking distance remains largely unaffected, the enhanced molecular ordering suggests stronger π–π interactions in P(NDICL-T2). These structural changes and the observed red shift in UV–vis absorption spectra from 700 nm to 732 nm suggest more ordered π–π polymer packing, likely facilitated by the cardanol substitution.

### 3.6. Electron-Transporting Properties

**Figure 5:**
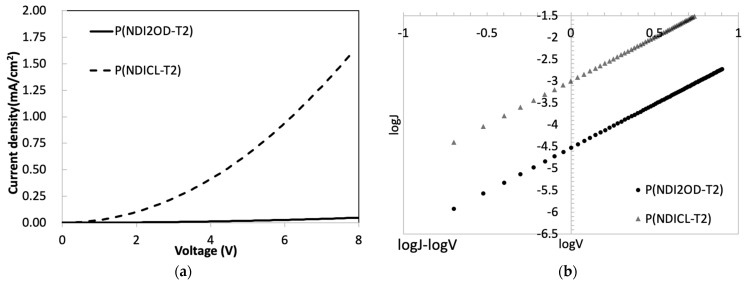
(**a**) Current density-voltage characteristics for electron-transporting devices based on P(NDI2OD-T2) and P(NDICL-T2); (**b**) double logarithmic plots characteristics for electron-transporting devices based on P(NDI2OD-T2) and P(NDICL-T2). Films were fabricated via the same procedure as UV-vis on 3.0 cm × 3.0 cm ITO substrates.

The electron mobilities of devices based on P(NDI2OD-T2) and P(NDICL-T2) are presented in Figure 5 and Table 2, showing a significant increase for P(NDICL-T2) relative to P(NDI2OD-T2). This improvement can be attributed to the substitution of the 2OD side chain with cardanol, which promotes more ordered packing in the thin-film state. As revealed by crystalline structure analysis using GIWAXD, P(NDICL-T2) exhibited additional diffraction peaks and a shift, indicating tighter lamellar spacing and enhanced molecular ordering. This enhanced ordering, along with the red shift in UV–vis spectra and reduced surface roughness observed in AFM images, suggests stronger π–π interactions facilitated by the cardanol substitution. These combined effects likely contribute to the observed increase in electron mobility for P(NDICL-T2).

## 4. Discussion

In summary, this work presents the successful synthesis and characterization of n-type polymers P(NDI2OD-T2) and P(NDICL-T2), incorporating cardanol-based side chains via Stille coupling. The substitution of the 2OD side chain with cardanol in P(NDICL-T2) led to substantial improvements in electron mobility, which can be attributed to enhanced molecular ordering and stronger π–π interactions facilitated by the unique structure of the cardanol side chains. UV–vis spectroscopy demonstrated a red shift in absorption for P(NDICL-T2), indicating more ordered π–π stacking and enhanced intermolecular interactions that favor efficient charge transport. Crystalline structure analysis revealed tighter lamellar spacing and additional diffraction peaks, suggesting enhanced molecular ordering in P(NDICL-T2). AFM analysis further supported these findings, revealing a decrease in surface roughness, which enhances film uniformity and contributes to the improved electron-transporting properties.

Overall, this study highlights cardanol as a promising side chain for optimizing the charge transport properties of n-type polymers, with potential applications in OFETs and OPVs. Future research could explore further structural modifications to optimize molecular packing and phase behavior, advancing the development of high-performance n-type semiconductor materials.

## Figures and Tables

**Table 1:** Characteristics of P(NDI2OD-T2) and P(NDICL-T2).

Polymer	*M*_n_ (g/mol) ^a^	PDI ^a^
P(NDI2OD-T2)	27,553	3.13
P(NDICL-T2)	24,346	3.76

^a^ Determined by GPC.

**Table 2:** Optical and electron-transporting properties of P(NDI2OD-T2) and P(NDICL-T2).

Polymer	*λ*_max_ (nm)	Electron Mobility (cm^2^/Vs) ^a^
P(NDI2OD-T2)	400; 700	2.95 × 10^−6^
P(NDICL-T2)	400; 732	1.01 × 10^−4^

^a^ Average values determined for four devices.

**Table 3:** Peak lattice spacing from GIWAXD (out of plane).

Polymer	*2θ* (*Degree*)			Lattice Spacing *d* (Å)		
	Alkyl	Lateral Packing	π–π	Alkyl	Lateral Packing	π–π
P(NDI2OD-T2)	3.5	-	22.5°	25.24	-	3.95
P(NDICL-T2)	5.2	17.5	22.5°	16.99	5.07	3.95

## Data Availability

The original contributions presented in this study are included in the article and Supplementary Material. Further inquiries can be directed to the corresponding authors.

## Supplementary Materials

The following supporting information can be downloaded at: https://www.mdpi.com/article/10.3390/mi15121475/s1, Figure S1: ^1^H-NMR spectrum of CL-2; Figure S2: ^1^H-NMR spectrum of CL-3; Figure S3: ^1^H-NMR spectrum of CL-4; Figure S4: ^1^H-NMR spectrum of NDICL-Br; Figure S5: ^1^H-NMR spectrum of P(NDI2OD-T2); Figure S6: ^1^H-NMR spectrum of P(NDICL-T2); Figure S7: GPC plots of P(NDI2OD-T2) and P(NDICL-T2).
