# Supplementary material for: Synthesis and Electron-Transporting Properties of N-Type Polymers with Cardanol-Based Side Chains
Source: Micromachines (Basel). 2024 Dec 5;15(12):1475. doi: 10.3390/mi15121475 (PMC11728016; doi:10.3390/mi15121475)
Supplement: Supplementary file 1 [file micromachines-15-01475-s001.zip › micromachines-3327586-supplementary.pdf]

# Supplementary Information

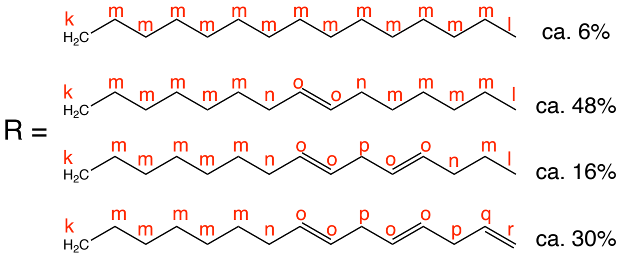


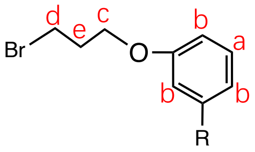


Structure of CL-2

$H_k = 2×6\% + 2×48\% + 2×16\% + 2×30\% = 2$
$H_l = 3×6\% + 3×48\% + 3×16\% + 0×30\% = 2.1$
$H_m = 26×6\% + 18×48\% + 12×16\% + 10×30\% = 15.12$
$H_n = 0×6\% + 4×48\% + 4×16\% + 2×30\% = 3.16$
$H_o = 0×6\% + 2×48\% + 4×16\% + 4×30\% = 2.8$
$H_p = 0×6\% + 0×48\% + 2×16\% + 4×30\% = 1.52$
$H_q = 0×6\% + 0×48\% + 0×16\% + 1×30\% = 0.30$
$H_r = 0×6\% + 0×48\% + 0×16\% + 2×30\% = 0.60$

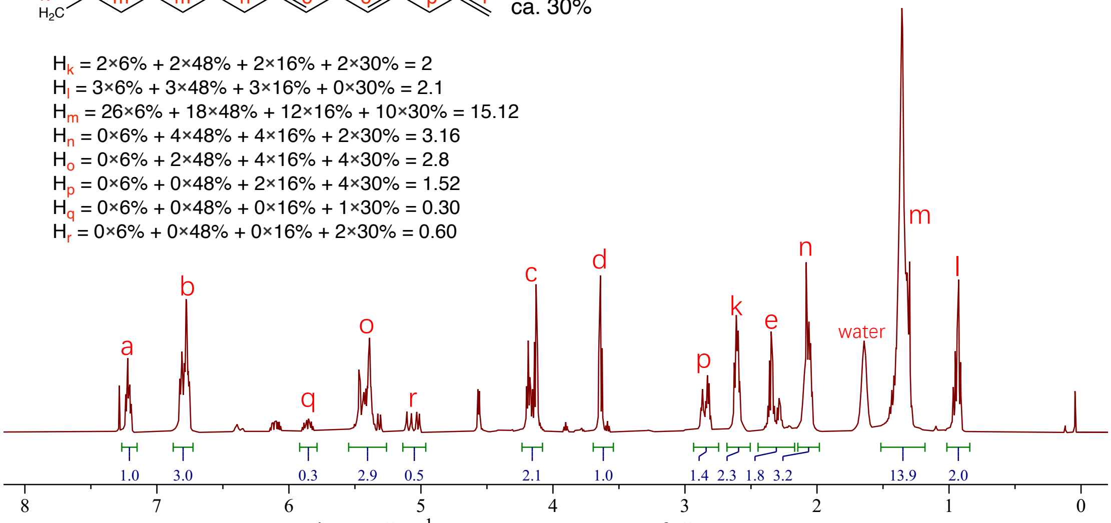


Figure S1: $^1$H-NMR spectrum of CL-2

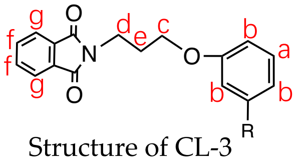


Structure of CL-3

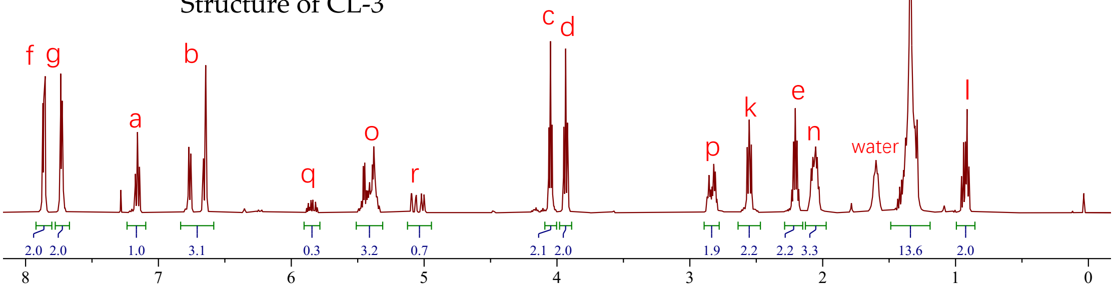


Figure S2 : $^1$H-NMR spectrum of CL-3

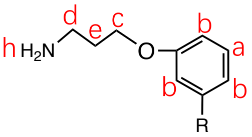


Structure of CL-4

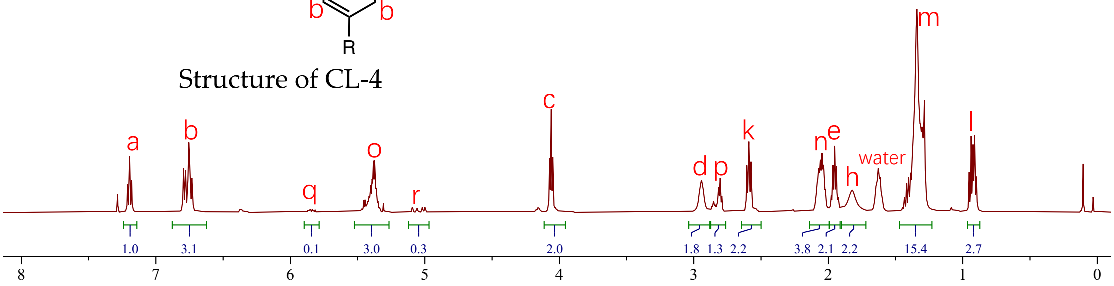


Figure S3: $^1$H-NMR spectrum of CL-4

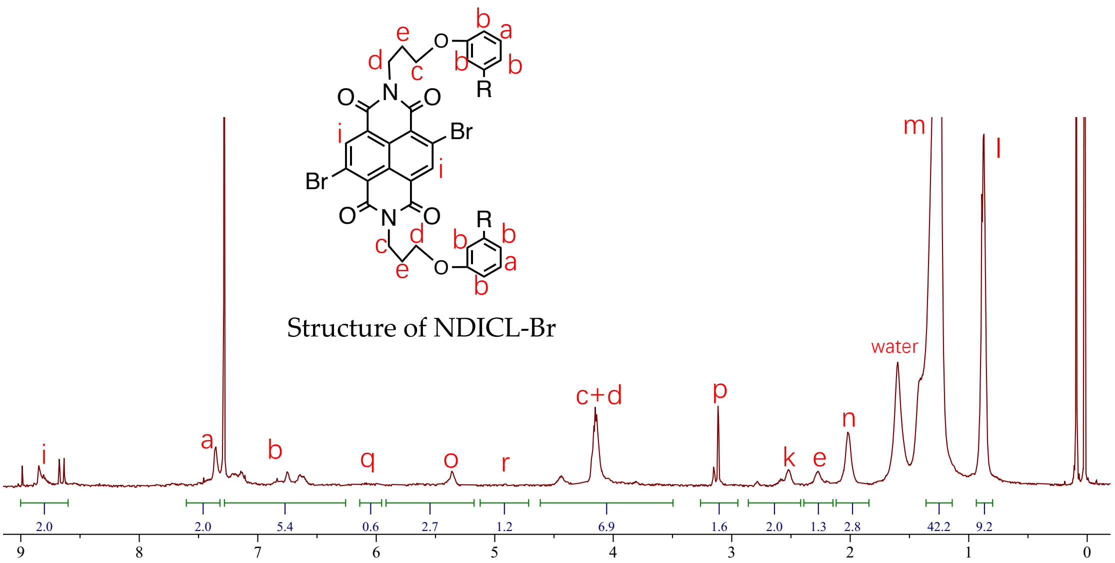


Figure S4: $^1$H-NMR spectrum of NDICL-Br

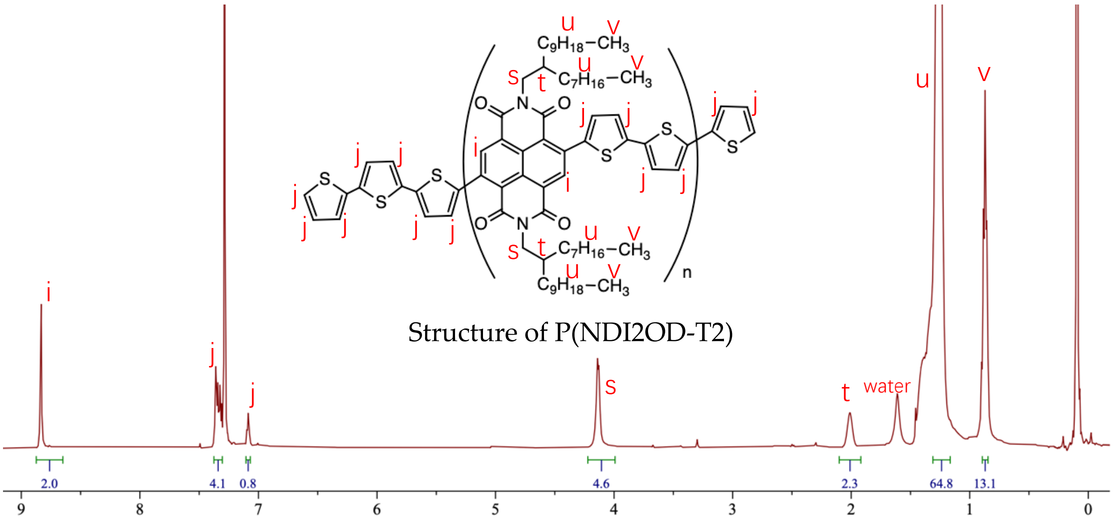


Figure S5: $^1$H-NMR spectrum of P(NDI2OD-T2)

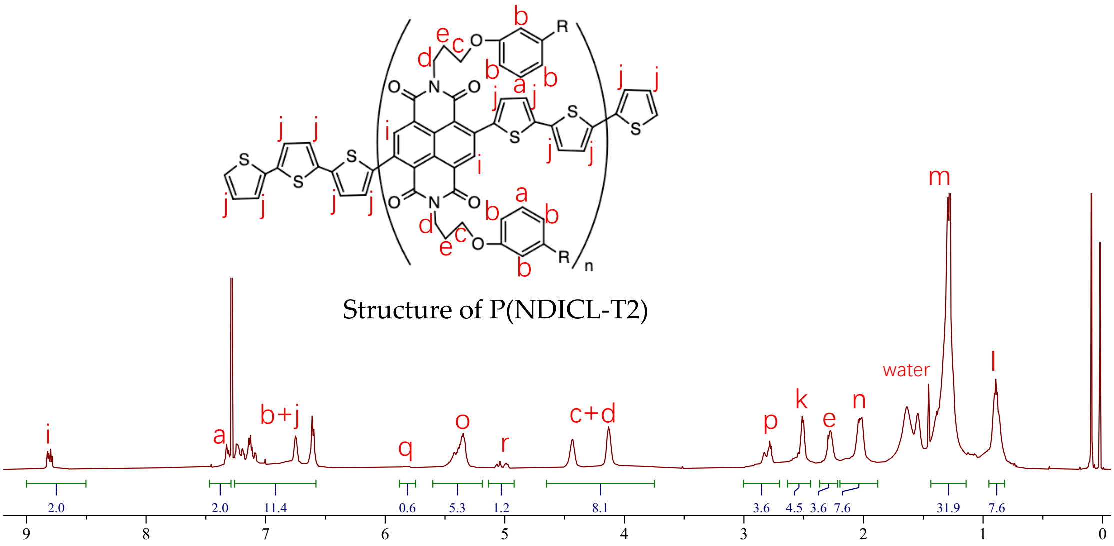


Figure S6: $^1$H-NMR spectrum of P(NDICL-T2)

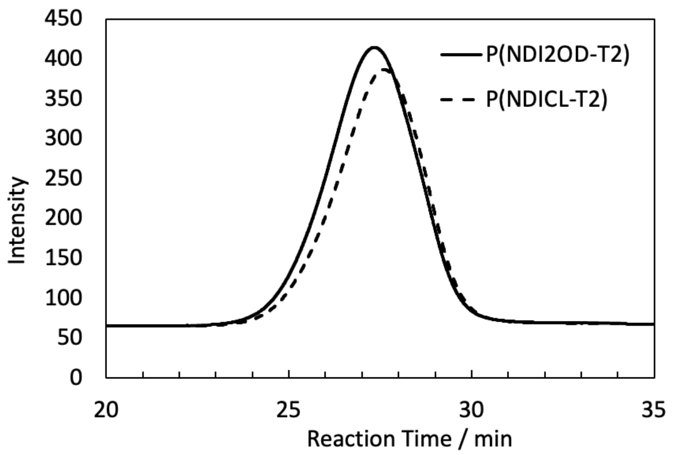

Figure S7: GPC plots of P(NDI2OD-T2) and P(NDICL-T2)
